# Federated Machine Learning, Privacy-Enhancing Technologies, and Data Protection Laws in Medical Research: Scoping Review

**DOI:** 10.2196/41588

**Published:** 2023-03-30

**Authors:** Alissa Brauneck, Louisa Schmalhorst, Mohammad Mahdi Kazemi Majdabadi, Mohammad Bakhtiari, Uwe Völker, Jan Baumbach, Linda Baumbach, Gabriele Buchholtz

**Affiliations:** 1 Hamburg University Faculty of Law University of Hamburg Hamburg Germany; 2 Institute for Computational Systems Biology University of Hamburg Hamburg Germany; 3 Interfaculty Institute of Genetics and Functional Genomics Department of Functional Genomics University Medicine Greifswald Greifswald Germany; 4 Computational BioMedicine lab University of Southern Denmark Odense Denmark; 5 Department of Health Economics and Health Services Research University Medical Center Hamburg-Eppendorf Hamburg Germany

**Keywords:** federated learning, data protection regulation, data protection by design, privacy protection, General Data Protection Regulation compliance, GDPR compliance, privacy-preserving technologies, differential privacy, secure multiparty computation

## Abstract

**Background:**

The collection, storage, and analysis of large data sets are relevant in many sectors. Especially in the medical field, the processing of patient data promises great progress in personalized health care. However, it is strictly regulated, such as by the General Data Protection Regulation (GDPR). These regulations mandate strict data security and data protection and, thus, create major challenges for collecting and using large data sets. Technologies such as federated learning (FL), especially paired with differential privacy (DP) and secure multiparty computation (SMPC), aim to solve these challenges.

**Objective:**

This scoping review aimed to summarize the current discussion on the legal questions and concerns related to FL systems in medical research. We were particularly interested in whether and to what extent FL applications and training processes are compliant with the GDPR data protection law and whether the use of the aforementioned privacy-enhancing technologies (DP and SMPC) affects this legal compliance. We placed special emphasis on the consequences for medical research and development.

**Methods:**

We performed a scoping review according to the PRISMA-ScR (Preferred Reporting Items for Systematic Reviews and Meta-Analyses extension for Scoping Reviews). We reviewed articles on Beck-Online, SSRN, ScienceDirect, arXiv, and Google Scholar published in German or English between 2016 and 2022. We examined 4 questions: whether local and global models are “personal data” as per the GDPR; what the “roles” as defined by the GDPR of various parties in FL are; who controls the data at various stages of the training process; and how, if at all, the use of privacy-enhancing technologies affects these findings.

**Results:**

We identified and summarized the findings of 56 relevant publications on FL. Local and likely also global models constitute personal data according to the GDPR. FL strengthens data protection but is still vulnerable to a number of attacks and the possibility of data leakage. These concerns can be successfully addressed through the privacy-enhancing technologies SMPC and DP.

**Conclusions:**

Combining FL with SMPC and DP is necessary to fulfill the legal data protection requirements (GDPR) in medical research dealing with personal data. Even though some technical and legal challenges remain, for example, the possibility of successful attacks on the system, combining FL with SMPC and DP creates enough security to satisfy the legal requirements of the GDPR. This combination thereby provides an attractive technical solution for health institutions willing to collaborate without exposing their data to risk. From a legal perspective, the combination provides enough built-in security measures to satisfy data protection requirements, and from a technical perspective, the combination provides secure systems with comparable performance with centralized machine learning applications.

## Introduction

### Background

Large data sets hold the promise of striking new insights by revealing even the faintest patterns that may, for example, be used to predict the most successful cancer treatment based on certain genetic markers. However, revealing these faint patterns often requires the use of machine learning—which in turn requires many large and well-prepared data sets as a basis for their training process [1]. Prediction models such as the one in our example are most effectively trained on patient data, that is, data that clearly relate to individual persons. This makes them “personal data” according to article 4 (1) of the General Data Protection Regulation (GDPR), which defines “personal data” as “any information relating to an identified or identifiable natural person” [2]. Collecting personal data, and especially aggregating it into large, centralized data sets, is fraught with substantial legal risks and often outright unlawful [3]. This is exacerbated by the legal “gray area” surrounding the lawfulness or unlawfulness of data collection in medical research. Such legal uncertainties inhibit the process of not only data mining but also, and especially, data sharing. Thus, the adoption of machine learning is inhibited [4]. Federated learning (FL), in which machine learning models are trained in a way that precludes the need for the aggregation of large data sets, is currently widely discussed as a possible technical solution to this problem.

We now introduce data protection regulation and privacy-enhancing technologies to show why a problem exists and provide the necessary background information.

### Data Protection Regulation

In many countries, machine learning systems need to comply with data protection regulations if they are intended to access and analyze personal data. Throughout the European Union, the GDPR has regulated this practice since May 25, 2018. Many countries have followed suit by launching similar legislation, partly to ease regulatory compliance when doing business with Europe [5-8]. The GDPR was introduced to address the potential for misuse that is inherent even in apparently innocuous personal data. Thus, these regulations were formulated with a broader perspective than medical data only. For instance, if the data collected about an individual by, for example, Google are processed and combined, they will likely provide an invasive view of that individual’s private affairs—even if each data item is innocuous in isolation. As such, there is no such thing as “irrelevant” personal data, and the collection of personal data always carries a risk to privacy and other fundamental rights [9]. This has always been the case for medical data, but the digitization of paper records introduces new challenges. The GDPR places a strict responsibility on the party controlling the data (according to the GDPR, the “controller”; see article 24) to ensure the protection of data “by design” and “by default” in article 25. The data processing does not have to be carried out by the controller themselves; the controller may use a processor “which processes personal data on behalf of the controller” (article 4 (8) of the GDPR). Such a “security paradigm,” in which the integrity, safety, and privacy of stored data are legally given top priority as design criteria for systems and processes, is not unheard of. For instance, medical information and other confidential matters are already dealt with only through highly secure systems. The importance of the GDPR lies in its expansion of the scope of legally protected data. “Personal data” is defined as “any information relating to an identified or identifiable natural person (‘data subject’)” by GDPR article 4 (1). These personal data fall under the requirements of the GDPR and are subject to stringent security and privacy requirements, as well as being difficult to exploit scientifically, commercially, or otherwise. Furthermore, the processing of genetic, biometric, and health data, which hold the potential for substantial medical discoveries, is placed under further restrictions (article 9 of the GDPR). The data subject has various rights in relation to personal data, such as the right to information, access, rectification, and erasure of their data. Only anonymous information is not subject to the GDPR [2], but a precise definition of “anonymous data” is not contained in the GDPR. Hence, it can only be deduced that anonymous data are the opposite of personal data, that is, data that contain no information related to an identifiable data subject [10]. In machine learning applications, the distinction between anonymous and personal data is very important because of its implications for the legality of data processing.

In the following section, we want to explore whether privacy-enhancing technologies are generally suitable for anonymizing (ie, eliminating the personal reference of) a data set and whether they can enable data to be processed in accordance with data protection requirements.

### Privacy-Enhancing Technologies

Privacy-enhancing technologies that are intended to allow for legally compliant processing and analysis of personal data include (1) FL, (2) differential privacy (DP), and (3) secure multiparty computation (SMPC). FL was proposed by McMahan et al [11] in 2016 as a potential solution to the conflict between data privacy protection and machine learning training [1].

**Figure 1:**
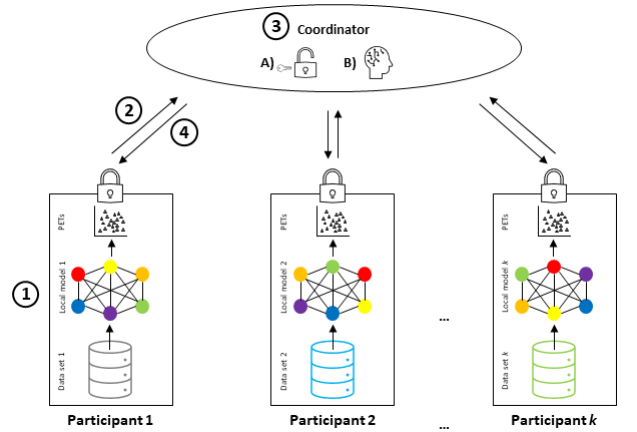
Schematic representation of federated learning combined with privacy-enhancing technologies (PETs). (1) Training of local models and implementation of PETs. (2) Secure transfer of local model parameters to a coordinator. (3A) Decryption of local models. (3B) Aggregation of local models. (4) Returning the global model to the participants. Note that these steps are often performed iteratively until model convergence (adapted from Huang et al [13]).

In FL, all data stay in their place of generation or storage and are never transferred to a central server, thus protecting the safety and privacy of the “local” raw training data [12]. FL includes at least 2 participants (eg, hospitals that provide data for a joint model), but each participant only has access to their own data. The process of FL is shown in Figure 1 [13]. After participants decide to collaborate, they engage in an iterative training loop, which has 4 steps (Textbox 1) that are represented by the circled numbers in Figure 1.

**The 4 steps in the iterative training loop.**

1. Training the local model
  - Each federated learning (FL) participant takes a baseline machine learning model and trains it on their own database, as in any other machine learning technique. They add privacy-enhancing technologies such as differential privacy (DP) or secure multiparty computation if necessary.
2. Securely transferring the local model to the coordinator
  - FL participants encrypt the (now trained) local model and send the encrypted local model to the coordinator (or a participant elected temporarily as a coordinator).
3. Aggregation of local models into the global model
  - The coordinator decrypts the local models of each FL participant and aggregates the parameters of each local model into a global model. DP can be implemented at this stage.
4. Return of the global model to the FL participants
  - The coordinator encrypts the global model and sends it to each participant. The global model, which contains the updates from the previous training round, becomes the “baseline” model for the new training round as the loop turns over to step 1.

Thus, in FL, participants perform model training only locally on their own data, whereas the generation of a federated global model is done by a coordinator who aggregates these local training results [12,14]. This process is often performed iteratively in several rounds until model convergence. For instance, FL’s application to digital health can enable insights to be gained across institutions collaboratively without the need to share personal patient data. Thus, the data never cross the firewalls of the institution where they reside [4]. The fact that data are never aggregated into a single central data set improves the quality of privacy protection and data security [1].

From a legal perspective, the quality of data and privacy protection that can be achieved through FL is of particular interest because of the aforementioned strict responsibility to ensure the protection of data “by design” and “by default.” Its workflow makes it uniquely suited for sensitive data such as health care data [15]. Nevertheless, a local model in an FL system can “leak” data about the training data set from the trained local model’s weights or parameters [16]. This is because FL systems are vulnerable to a number of attacks, for example, privacy leaks during data communication [3,17,18] through predictions based on the model and background information [17,19] or poisonous attacks by malicious clients [3,18,20-33]. As such, FL is typically combined with other privacy-enhancing technologies [34,35]. In this review, we discuss DP and SMPC as potential solutions. DP can reduce the risk of data leakage by adding noise to the training process, which makes it more difficult to make inferences about the underlying patient data [15,16]. “DP can be applied to the input data (local DP), the computation results (global DP) or the algorithm” [36]. There is a trade-off inherent to DP as “adding randomness to the collected data preserves user (participant) privacy”—the main objective in terms of data protection on legal compliance—“at the cost of accuracy”—the main concern in terms of creating a useful machine learning application [16]. In the end, “the goal is to achieve an optimal balance between privacy and result quality” [37]. In addition to the intrinsic quality-privacy trade-off, DP cannot eliminate but only reduce data leakage risks [16]. Another privacy-enhancing technology that can be integrated into FL systems is SMPC. If implemented correctly, SMPC allows “multiple parties [to] collaborate to compute a common function of interest without revealing their private inputs to other parties” [16]. The challenge is to reveal as little information as possible to any given counterparty as the other parties may potentially collude to piece together disparate pieces of information revealed during computation [38].

In this scoping review, we pursued the following two objectives: (1) provide an overview of the current literature assessing the legal aspects of FL and the privacy-enhancing technologies DP and SMPC relevant for medical data and (2) illuminate and specify unsolved legal challenges and provide recommendations for action for clinicians and researchers in the medical field.

For a better overview and structure, we defined the research questions (RQs) outlined in Textbox 2.

**Research questions (RQs).**

**RQ 1**

- Are the local or global models used in federated learning (FL) “personal data” as defined in article 4 (1) of the General Data Protection Regulation (GDPR)? What could be the consequences in legal terms?

**RQ 2**

- What are the roles of FL model service providers and training participants in relation to the controller and processor roles set out in chapter 4 of the GDPR?

**RQ 3**

- Who controls the (raw) training data used to train the local models in decentralized FL? Who controls the model updates aggregated into the global model?
- RQ 3.1
  How are the raw training data and model updates secured?
- RQ 3.2
  Which processing bases, basic principles of data protection law, and rights of data subjects must be observed during model training?

**RQ 4**

- Does the use of secure multiparty computation and differential privacy change the legal assessment of FL?

## Methods

We performed a scoping review according to the PRISMA-ScR (Preferred Reporting Items for Systematic Reviews and Meta-Analyses extension for Scoping Reviews) [39].

### Eligibility Criteria

To cover a wide variety of publications, we included any publications dealing with FL and its legal aspects. Hence, we did not set any limitations on the source of literature and included published books, scientific papers, industry publications, and matters of public record. We only looked for literature published after January 1, 2016, as FL was first introduced in that year [11]. Finally, for reasons of transparency and practicality, the included publications needed to be open access and written in English or German.

Publications unrelated to our main topics (ie, our RQs) or to FL and legal aspects or that addressed only one of both topics were excluded.

### Information Sources

We searched for literature in the search engines and databases Beck-Online, SSRN, ScienceDirect, arXiv, and Google Scholar, including PubMed. The searches were performed between January 13, 2022, and February 18, 2022.

### Search

In the selected databases and search engines, we first tried various search string criteria. We looked for synonyms of “Federated Learning,” our key term, and identified “FL,” “Federated Machine Learning,” and “Federated ML.” Similarly, we considered and looked for synonyms of our second key term, “Data protection,” and found “Data privacy protection,” “GDPR-Compliance,” “privacy protection,” “Differential privacy,” and “Secure Multiparty Computation.” Hence, in our searches, we combined “Federated Learning” AND “Privacy Protection.” Synonyms were added to the main term using OR. Finally, we applied the aforementioned publication year restrictions. Thus, the final search in, for example, Google Scholar was “((federated learning) AND ((data protection) OR (privacy protection) OR (GDPR-compliance) OR (DSGVO)) AND (Years 2016-2022 [Filter])).” The search strings finally selected to obtain the most useful information terms and the respective number of publications in the first search of each search engine are listed in Tables S1-S5 in Multimedia Appendix 1.

### Selection of Sources of Evidence

The literature selection was performed independently by 2 reviewers (AB and LS), and disagreement was resolved via discussion. In the first step, AB and LS screened the titles and abstracts for inclusion; afterward, they evaluated the full texts of the preselected publications.

### Data Charting Process

Although we screened the full texts for inclusion, we observed a large heterogeneity in the publication structure. Furthermore, we found that most publications only addressed 1 or 2 of our RQs. As a consequence, we refrained from a structured data extraction with, for example, a spreadsheet. Instead, to ensure that all relevant information was respected, 2 reviewers (AB and LS) independently extracted data from the included publications. Afterward, we clustered these data according to our RQs.

### Data Items

For each of the included publications, we documented the author, publication year, title, and data source from which we obtained it. Finally, we also registered which of our RQs was addressed by the publication.

### Critical Appraisal of Individual Sources of Evidence

We did not evaluate the quality of the included information sources. However, as we only searched for and included publications identified by data engines and databases for scientific purposes, a minimum of quality was guaranteed. Furthermore, it should be noted that, although medical publications are often peer-reviewed before publication, this is not standard for law-related publications. A review of these publications is conducted by the internal editorial board of the journals. Finally, we only considered open access publications to allow our curious readers to evaluate the quality of the included publications for themselves.

### Synthesis of Results

After we extracted relevant information from the publications, we grouped it in accordance with our RQs. The extracted information was added to one or more of our RQs if relevant. Finally, we cross-checked whether all the extracted information was covered in our results. Afterward, we quantified the agreement between publications regarding our proposed RQs.

## Results

### Search Strategy

**Figure 2:**
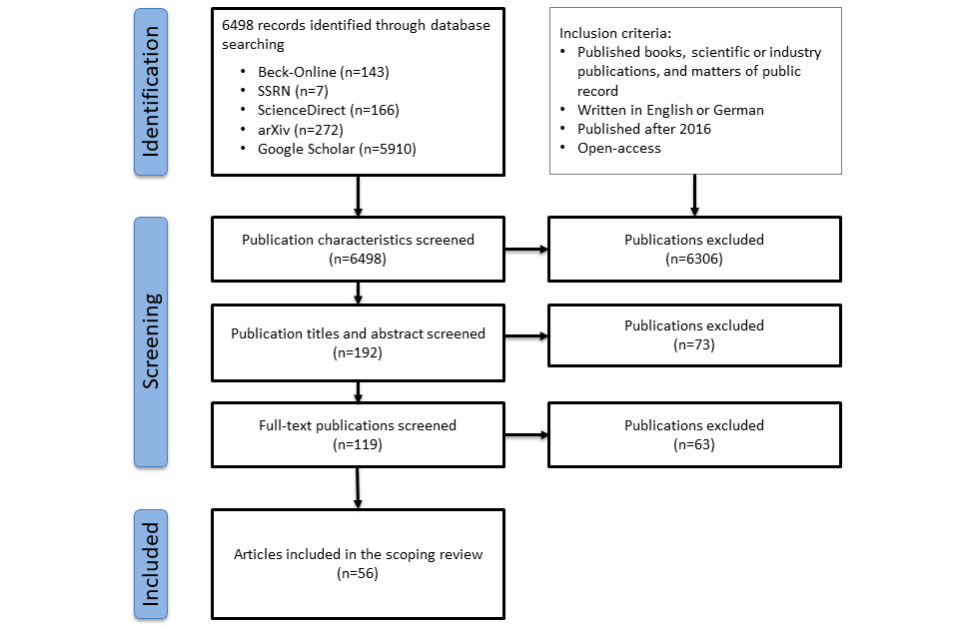
PRISMA (Preferred Reporting Items for Systematic Reviews and Meta-Analyses) flow diagram showing disposition of publications. Of a total of 6498 initial articles, 56 (0.86%) were eligible for data abstraction. The most common source of the selected articles was Google Scholar, followed by arXiv.

The flowchart in Figure 2 provides an overview of our publication inclusion and exclusion processes. Of the identified 6498 publications, 56 (0.86%) fulfilled our criteria and were included—55 (98%) articles and 1 (2%) book. Most of the articles were published in 2020 (20/56, 36%) and 2021 (24/56, 43%).

Most of the identified studies (50/56, 89%) were limited to either only the legal or technical dimension of data protection in FL. Thus, we could only include 11% (6/56) of studies [3,22,28,40-42] that made a direct link between the 2 disciplines.

An overview of the included publications, including which publications addressed which RQ, is provided in Table S1 in Multimedia Appendix 2 [1,3,17-34,40-75].

In the following sections, we present our results as they correspond to our RQs. The 2 most important papers were Rossello et al [28], “Data protection by design in AI? The case of federated learning,” and Truong et al [22], “Privacy preservation in federated learning: An insightful survey from the GDPR perspective,” both of which discussed all of our RQs. RQs 2 and 3 were each only addressed in a total of 5% (3/56) of the publications. Most studies (4/56, 7%) were published in the past 3 years (2020-2022), highlighting an emerging area of interdisciplinary study relevant to clinical researchers. Further research in this area can be expected—particularly as judicial decisions and new regulations begin to arrive and the ecosystem of FL tools available to medical researchers grows in scope, performance, and availability.

### RQ 1: Are Local or Global FL Models “Personal Data” as Defined in Article 4 (1) of the GDPR? What Could Be the Consequences in Legal Terms?

Personal data are information related to an identified or identifiable natural person. Patient data fulfill this requirement. Training data in FL systems stay at their place of origin or production and cannot be viewed by the central party at any time. Of the 56 included publications, 37 (66%) addressed this question and 18 (32%) agreed that FL leads to better privacy and security compared with centralized systems [3,17,19,21-23,26,28,29,31,32,40,43-48].

Two contradicting views are held regarding our first RQ: (1) local models are sufficiently anonymized to not constitute personal data and (2) local models are personal data. According to the first view, local models should not be considered personal data [3] as the coordinator responsible does not have access to the training data, only to the trained models. These can be considered not personal data as several processing steps occur before transmission, “which individually are already suitable for eliminating the personal reference of the models, and even more so in the aggregate.” Thus, the models that are shared should typically qualify as anonymous [40]. This allows the local models “to be processed without restrictions imposed by the GDPR” [3].

According to the other view, the local model as constituted by the number vector containing the parameters that result from training the model on the local data, which is shared with the coordinator, can be considered personal data unless privacy-enhancing measures are used [28,41]. This is due to the aforementioned “data leakage” risks [3,18,20-31,33,49-55]. “Some features of the training data samples are inherently encoded” [22,34,56,57] into local models as the training participants “represent diverse users (e.g. patients) with different interests, preferences and habits” [58]. Therefore, “the underlying data distributions of the users are not identically distributed and as a consequence, is characteristic of the users” so that model updates encode individual-specific information (acting as a fingerprint) [58].

Although the global models themselves are considered to be anonymous and, therefore, not personal data [22,23,41], the GDPR is still applicable to local models and model updates [3,22,28,40,43,45]. In this case, the “controller(s) responsible for the processing operations on these data will have to ensure that the processing of model updates complies with the GDPR” [28]. Therefore, it is necessary that “the processing rests on one of the legal grounds listed in articles 6.1 juncto 9.2 GDPR and that the purpose of the training is compatible with the purpose for which the data were originally collected, pursuant to articles 5.1.(b) and 6.4 GDPR” [28]. It is also assumed that the processing operations performed on personal data in FL are likely to fall under the definition of “processing” under article 4 (2) of the GDPR [28].

Overall, these contradictory opinions could lead to practicians and hospitals being “more reluctant to participate in FL without proper privacy protection” [49].

### RQ 2: What Are the Roles of FL Model Service Providers and Training Participants in Relation to the Controller and Processor Roles Set Out in Chapter 4 of the GDPR?

**Figure 3:**
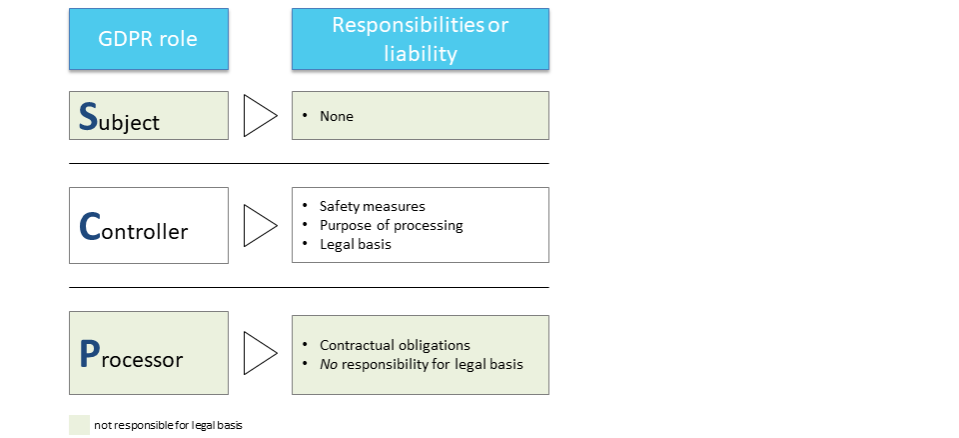
Representation of the roles that are laid out in the General Data Protection Regulation (GDPR). These are combined with their respective responsibilities or liabilities as defined by the GDPR.

In the GDPR, there are 3 participant roles (Figure 3): data subject (article 4 (1) of the GDPR), data controller (article 4 (7) of the GDPR), and data processor (article 4 (8) of the GDPR) [22,28]. The data subject (eg, a patient) is the identified or identifiable person; they have the right to access, erasure, and restriction of processing [22]. The data controller (eg, a hospital or a clinician) determines the processing purposes in accordance with the GDPR and ensures the privacy and security of the data [22,28]. The controller has to inform the data subject about the sharing and processing of their data [22]. However, first, the data controller must ensure the existence of a legal basis, that is, a legitimation, for data processing (article 6 of the GDPR), most importantly by obtaining the data subject’s consent (article 6.1 (a) of the GDPR) [22]. The data processor (eg, the quality management department of the hospital or a researcher) processes the data for the purposes laid out by the controller [22]. However, in FL, there is an additional role: the FL participant.

**Figure 4:**
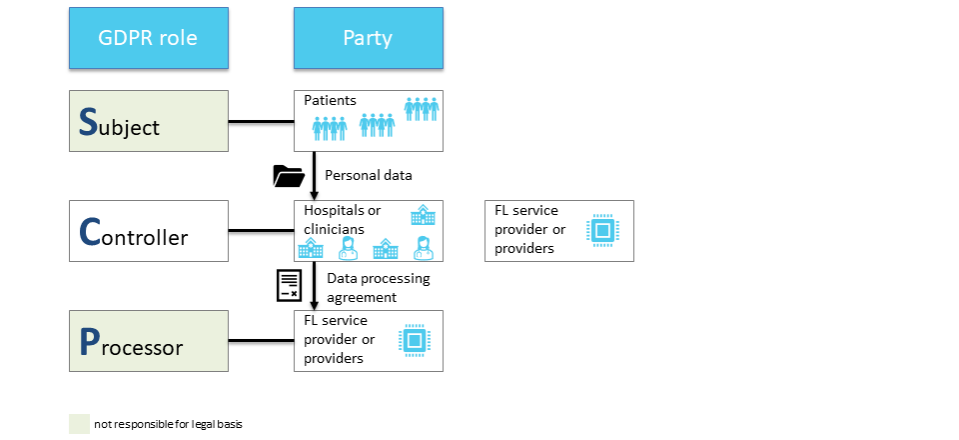
Representation of the relationships between the different parties and the roles they fulfill, including the “FL Service Provider.” FL: federated learning; GDPR: General Data Protection Regulation.

The service provider of an FL model fulfills the roles of both data controller and data processor but not those of other players (ie, third parties, as defined in article 4 (10) of the GDPR) [22,28] (Figure 4). They implement the FL system; direct the participants (eg, hospitals and clinicians) to train, share, and update their (locally trained) models; and aggregate and update the global models to all participants [22]. Note that the FL service provider is likely to only have contact with the data subjects via the participant—hence, there is no direct contact. Owing to the concept of joint control and the complexity of many FL systems (eg, potentially millions of patients providing raw training data), the service provider is not necessarily the only data controller [28]. Therefore, it can be challenging to identify and allocate the responsibility of each actor for compliance with the GDPR, in particular toward data subjects [28]. Inaccuracies could violate the principle of transparent and fair processing laid down in article 5.1 (a) of the GDPR [28].

**Figure 5:**
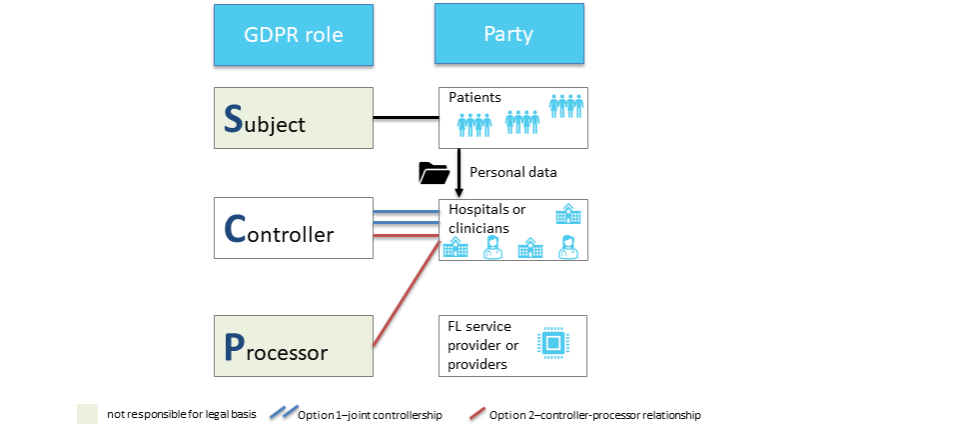
Representation of the relationships between the different parties and the roles they fulfill. The role of the service provider(s) is not depicted. The figure shows the 2 options of joint controllership (option 1) and a controller-processor relationship (option 2), which can be chosen by the participants. FL: federated learning; GDPR: General Data Protection Regulation.

Regarding the roles of (potential) FL participants, it will be necessary to examine “whether the relation between training participants is qualified as a controller-processor or a (joint) controllership relationship (article 26 GDPR)” [28] (Figure 5).

Moreover, and regardless of the outcome, each training participant should “conduct a careful due diligence investigation” of *all other parties’* compliance with the GDPR “before venturing into a federated learning scheme” [28]. The question of whether such a process can realistically be undertaken by all participants is beyond the scope of this study.

### RQ 3: Who Controls the (Raw) Training Data Used to Train the Local Models in Decentralized FL? Who Controls the Model Updates Aggregated Into the Global Model?

#### Overview

**Figure 6:**
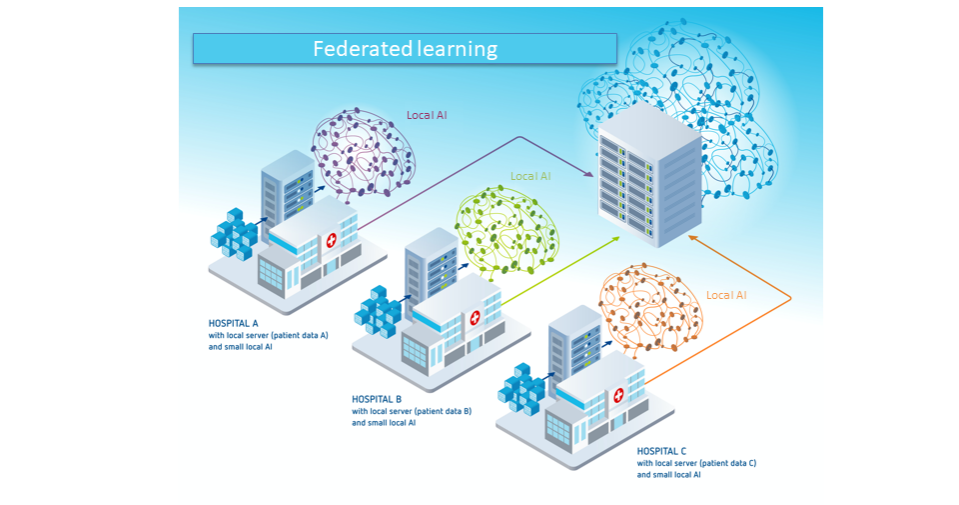
Schematic representation of federated learning with hospitals as participants without differential privacy and secure multiparty computation (adapted from FeatureCloud [59], with permission from the FeatureCloud consortium).

The training data are provided by contributors (referring to Figure 1, this would be participant 1 to participant *k*) who update the local models themselves and are the only party that controls their respective data [22,28]. The FL developers [21] and service providers [22] are not able to access the training data. Model updates are generated by the machine learning system on the local system and transferred to the coordinator—hence, unlike the raw data, they change hands throughout the training process (Figure 6). This process creates a technical data protection risk, at least in the absence of encryption. It also creates substantial legal risk insofar as local models may be in fact personal data.

#### RQ 3.1: How Are the Raw Training Data and Model Updates Secured?

Training data and model updates can be secured using privacy-enhancing technologies such as data anonymization, DP, SMPC, and homomorphic encryption [22,60,61]. Which technology or combination is the most effective depends on the respective circumstances and type of data (eg, genetic data cannot be successfully anonymized).

#### RQ 3.2: Which Processing Bases, Basic Principles of Data Protection Law, and Rights of Data Subjects Must Be Observed During Local Model Training?

**Figure 7:**
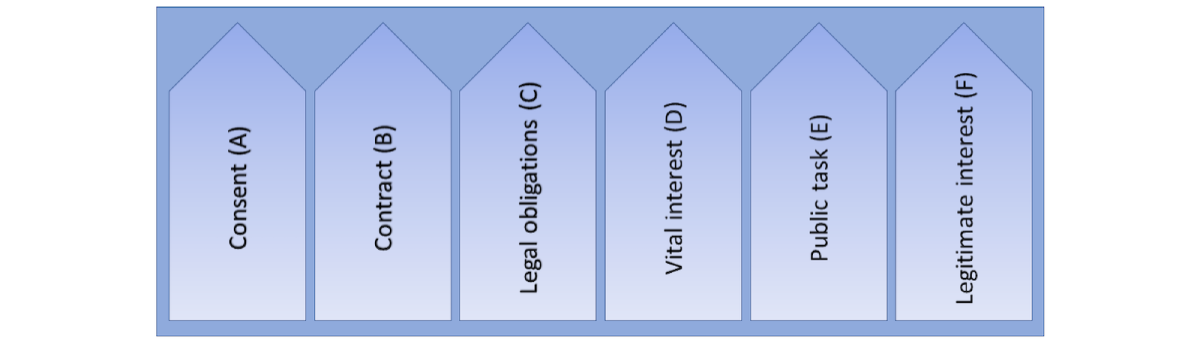
Processing bases (article 6.1 of the General Data Protection Regulation [GDPR]). At least one of the 6 requirements (processing bases A-F) according to article 6.1 of the GDPR must be met for personal data to be processed lawfully (this does not pertain to personal data that fall under a special category according to article 9.1 of the GDPR, such as health or genetic data).

There are 6 data processing bases determined in article 6.1 of the GDPR (Figure 7).

The controller is responsible for ensuring that one of the processing bases applies. In practice, the underlying bases of *consent* (article 6.1 (a) of the GDPR) and *legitimate interest* (article 6.1 (f) of the GDPR) are especially important [3,22].

*Consent* (article 6.1 (a) of the GDPR) may be considered “but will not usually be given by data subjects [patients]; after all, it is voluntary” [3]. “The training of models is also not *necessary* for the *performance* of a contract (Art. 6.1 (b) GDPR), which would be the case, for example, with the processing of account data for salary payments” [3]. In practice, there is a presumption that companies will frequently invoke the so-called overriding legitimate interests pursuant to article 6.1 (f) of the GDPR [3]. A legitimate interest in accordance with article 6.1 (f) of the GDPR exists “if it is necessary to process the data due to legitimate interests of the controller and the data subjects’ interests, fundamental rights and freedoms regarding the protection of their personal data do not outweigh the controller’s interests” [3]. Relevant aspects of this weighing are the amount of data processed, applied security mechanisms, data access, content, and purpose of the individual case [3]. This means that the GDPR does provide a way to process personal data beyond the minimum processing required to fulfill a contract but only if the strictest security is maintained. Article 9.2 of the GDPR opens up the processing of sensitive data such as health, biometric, or genetic data in specified cases. Included are consent (article 9.2 (a) of the GDPR) and scientific research purposes (article 9.2 (j) of the GDPR).

Data minimization requires the controller (eg, service provider) to only collect data that are adequate, limited, and relevant exclusively to the agreed-upon purposes [22]. Purpose limitation requires data subjects to be informed of the purpose of the data collection and limits the use of the data to the initially expressed or compatible purposes [22,28]. Storage limitations require data to be anonymized or deleted after having fulfilled their purpose. Medical records may mostly be deleted 5 to 10 years after the last discharge or after death. The principle of accuracy requires that the stored data be correct and updated. Largely, FL systems easily comply with most of these requirements [22] except fairness and transparency [22].

For both data minimization and purpose limitation, privacy measures to protect the data from unauthorized access and extractions must be taken [22]. However, FL systems generally comply with these principles because of their architecture [22]. Similarly, FL systems meet accuracy requirements as they only process model parameters in their original and unaltered form [22] unless the model performance is impaired owing to poisoning attacks [28]. As “the ‘raw’ training data provided by each FL participant can ‘by design’ not be inspected by other actors than the holder of the data,” no participant can guarantee that other participants comply with the principles of accuracy [28]. FL systems inherently comply with the principle of storage limitation as they only store the global models that do not contain personal data [22]. As for integrity and confidentiality, as FL systems cannot guarantee the privacy of the raw data, additional techniques have to be deployed by coordinators’ servers but also by FL participants [22]. As for fairness and transparency, GDPR compliance also requires fairness and transparency, which FL systems do not completely fulfill [22,28,44]. If the training data are not carefully and correctly collected, biased results can lead to discrimination and injustice [22], which, in terms of medical research, could mean poorer health care for certain populations. Furthermore, practices and mechanisms designed to assure data privacy, including the inability of service providers to access the raw training data and local models (which, as mentioned, serves among other things to fulfill the principle of purpose limitation), prevent FL systems from complying with these principles [22]. These issues are fundamental to FL and machine learning in general. Regarding the rights of the data subject, it is worth mentioning that the training process in FL as a whole is automated within the meaning of article 22.1 of the GDPR [22,23]. As a consequence of the so-called black box effect, which is generally inherent in machine learning, including FL, there is limited transparency regarding the training process and the results (eg, a global machine learning model in FL), which “are generally generated without any proper explanation” [22]. This causes uncertainty about whether outcomes of a machine learning model have a negative impact on the data subject (eg, patient) “or negatively impact its circumstances, behavior or choices” [22]. Compliance with the GDPR remains possible if data subjects explicitly consent to relinquishing their rights to control automated decision-making [22]. Other, more remote possible solutions to these issues are the development of new machine learning techniques or the relaxation of current regulations [22].

**Figure 8:**
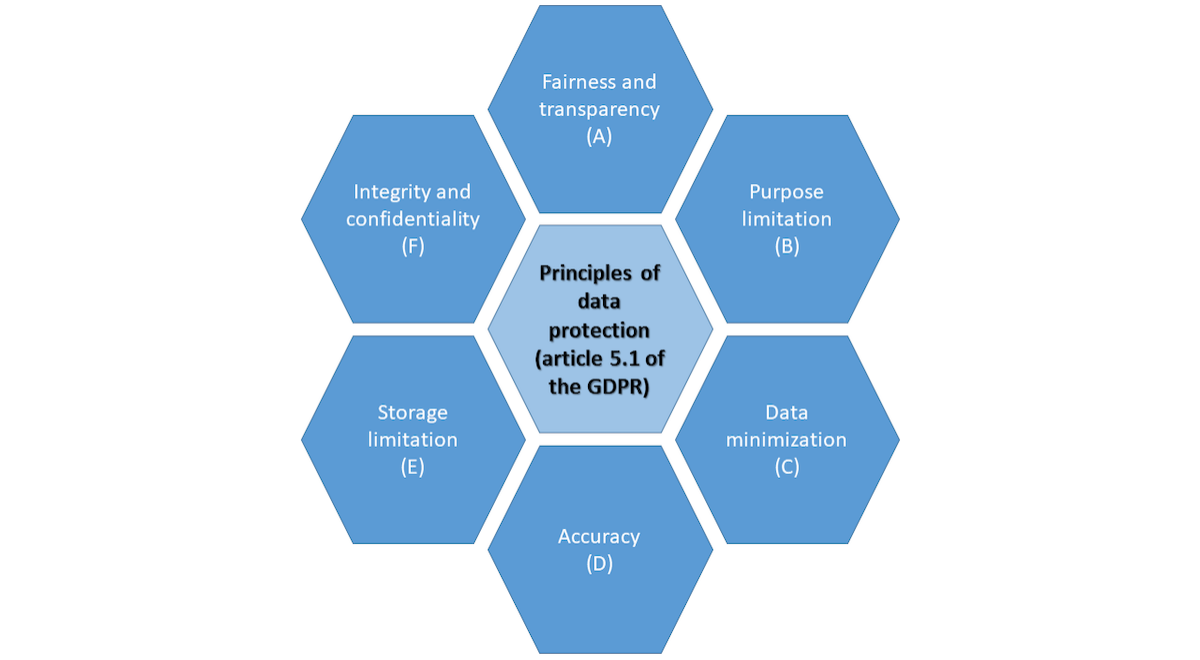
Principles of data protection (article 5.1 of the General Data Protection Regulation [GDPR]). All 6 principles of data protection according to article 5.1 of the GDPR (A-F) must be followed to ensure lawful data processing.

In addition, the system must comply with basic principles of data protection (Figure 8).

### RQ 4: Does the Use of SMPC or DP Change the Previous Legal Assessment?

RQ 4 was addressed in 45% (25/56) of the publications. Fundamental to our last RQ is the recognition that FL does not fully guarantee the privacy of personal data on its own [22]—FL offers a way to make the training of artificial intelligence (AI) models more secure in terms of data protection law and, therefore, more attractive for those involved, but other privacy-enhancing measures must be taken to protect personal data [42]. If the anonymity of a model cannot be guaranteed, the GDPR applies, and controllers are required to fulfill their obligations as set out in the GDPR, starting with providing a justification for data processing that provides a legal basis according to articles 6 and 9 (see the previous sections) [41]—2 views differing in their assessment of the value of privacy-enhancing technologies. According to the first view, the privacy-enhancing technologies explored previously are sufficient to overcome the weaknesses of FL on its own. According to the other view, DP and SMPC as a privacy enhancement for FL need to be substantially redesigned to provide data subjects with a meaningful degree of data protection.

**Figure 9:**
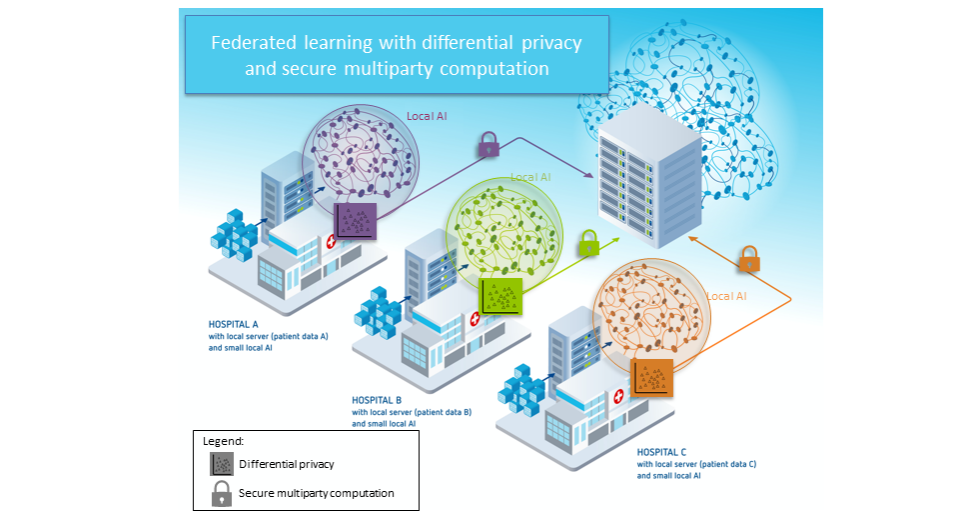
Schematic representation of federated learning with hospitals as participants combined with differential privacy and secure multiparty computation (adapted from FeatureCloud [59], with permission from the FeatureCloud consortium).

Article 25 of the GDPR “takes into account the realization that adequate protection of privacy in the digital age is inconceivable without ‘privacy by design’” [62]. Hence, for data processing technologies and procedures, privacy must be fundamental at all levels of programming and architectural design and must be ensured from day 1 of development [62]. This can be achieved by integrating privacy-enhancing technologies such as DP into the processing operations [62] (Figure 9). In this context, some argue that the combination of FL with other privacy-enhancing technologies such as DP “limits the capacity to extract the (personal) training data from the [local model] updates sent to the coordinator” [28,50,63,64]; others even hold that the system becomes fully private when DP and SMPC are combined [65].

It is also said that, as the effort required to extract personal data from global models increases with every privacy-enhancing measure taken, combining privacy-enhancing technologies such as DP and SMPC allows the global model and its process of aggregation to qualify as anonymous*—*and, hence, not fall under the scope of the GDPR [41].

If one nevertheless considers a given process to fall within the scope of the GDPR, it is worth mentioning that, in the context of weighing of interests according to article 6.1 (f) of the GDPR, the processing of personal data in FL leads to “an overriding on the part of the provider [data controller], as long as the provider ensures through appropriate measures in accordance with the state of the art that access by third parties is practically excluded” [3,40].

It is difficult to extract a consensus from the literature, particularly as the terminology is not quite settled. For instance, in 1 source, DP is touted as a means to overcome the transparency issue—something that the DP techniques we have previously described definitely cannot do and whose possibility in the first place is not even certain [66].

According to the other view, these problems are too fundamental. Even within the FL framework, it is not necessarily possible to “avoid algorithms recording sensitive data or even learning to discriminate” [33]. It is also argued that privacy-friendly techniques such as DP, which are designed “to prevent unintentional disclosure of sensitive data” in accordance with article 25 of the GDPR, do not guarantee that the data are free of “errors” [67]; therefore, the question of the compatibility of FL in combination with privacy-enhancing technologies with the basic principles of the GDPR is raised again (see the previous sections). Furthermore, it is also argued that combined privacy-enhancing technologies cannot guarantee that the system is fully private [68].

In addition, a malicious coordinator puts DP implementations at the coordinator level at risk “as they explicitly trust the central party with the crucial task of adding DP noise, and thus provide no protection against a malicious central party” [69]. Moreover, adding noise at the global scale severely harms the utility of the model as it reduces the accuracy of the trained or central data set [54,57,69-76]. This, in turn, contradicts the principle of accuracy (article 5.1 (d) of the GDPR). As such, for FL to provide meaningful privacy protection to participants and fulfill the principles of the GDPR, a considerable redesign is required [69].

## Discussion

### Principal Findings

This review explored the potential and shortcomings of FL in terms of privacy and data security with a focus on medical data. The major identified problem is defining the GDPR status—personal or anonymized data—of which only the former is governed by the GDPR. We found that, in addition to the data themselves, the GDPR status of both local and global FL models is uncertain. Without DP and SMPC, local FL models should be considered personal data and, thus, need to be treated as such. Moreover, there is controversy as to whether DP and SMPC are sufficient to “anonymize” local models. Whether global models are personal data is also uncertain. Therefore, in general, it remains unclear whether FL achieves a level of privacy and security consistent with the requirements of the GDPR. Although FL systems do provide better security than centralized systems, they do not by themselves ensure a sufficient degree of anonymization and privacy to be considered GDPR compliant by design. Thus, even if global models are not to be considered personal data, the GDPR remains applicable to local models and model updates.

In the following sections, we will explore the open questions we identified as requiring further regulatory action. The GDPR circumscribes the conditions under which the processing of personal data is possible. However, further refinement is needed as the *concrete* requirements for FL service providers as set out in the GDPR are currently very unclear [28]. This lack of legal certainty for FL providers is likely to obstruct the adoption of FL technologies despite their potential for solving major legal issues. A particularly fatal example of this problem is the lack of criteria for differentiating personal and anonymous data that can be used to determine the status of data beyond doubt and without requiring recourse to legal experts [37]. In the absence of such verifiability, there is no guarantee that a data set anonymized according to the state of the art is *truly* anonymous and, thus, whether the GDPR applies. This raises 2 interlinked questions. The first is what degree of anonymity is sufficient in each case and also in relation to the various types of data. The second is whether the anonymization used makes the data anonymous and whether this anonymity is resilient to attacks [37]. Furthermore, the terms *personal* and *anonymous*, which we have used throughout this paper, should be furnished with a precise legal definition that allows for the evaluation of data without recourse to jurists [37]. This is a tricky problem for policy makers to solve, not least as progress in the development of cryptographic and analytical techniques is likely to affect the suitability of various anonymization techniques going forward. Until there is a settled jurisprudence on this question, FL service providers will navigate an environment of substantial legal risk.

### Applicability of Privacy-Enhancing Technologies

Several privacy-enhancing technologies exist, but they cannot be applied in all cases [37]. Applying privacy-enhancing methods to arbitrary machine learning methods is difficult and often impossible as they are optimized for application with specific learning algorithms [37]. In addition, “lack of scalability is an obstacle to applying privacy-enhancing measures in practice” [37].

Data protection, particularly if pursued at the high level mandated when processing personal (medical) data, always generates costs. This is due to the higher computational effort, longer training times, and reductions in the utility of the data, for example, through added noise [37]. DP adds noise to the data to ensure no identifiability of local data from trained models but faces the challenge of balancing privacy levels and model utility—the more noise, the more privacy but the lower the accuracy of the model (although this is a problem with DP in general independent of the application to FL) [47]. These costs at the model level of anonymity may mean that an application that is valuable in principle is not sustainable in practice [37].

### Technically Challenging GDPR Obligations

Some requirements of the GDPR seem downright impossible to fulfill for FL applications. The first is the requirement for error-free data sets, and the second is the requirement for transparency. For this reason, some refinements to the GDPR have already been mooted or are in progress of being delivered.

The requirement for error-free data sets comes from the principles of lawfulness, fairness, and transparency set out in the GDPR but presents an almost impossible challenge [77]. The reason for this is that the vast amounts of data used in machine learning systems cannot feasibly be verified in their entirety. In FL systems, the data are distributed among many FL participants who, by design, cannot access each other’s data, which exacerbates the problem of verifiability of data. In consequence, regulating the *process* of validating data sets rather than mandating the *outcome* of completely error-free data sets is recommended [67]. In addition, measures to evaluate the quality of data sets should be established (“e.g., predictive accuracy, robustness, fairness of trained machine learning models”) [67]. Owing to the difficulty of verifying other participants’ regulatory compliance in an FL collaboration, it may be necessary to implement ex ante *accountability measures*, “particularly, those concerning the ‘quality’ of the training data” [28]. These measures should act as a basis for each training participant to be able to demonstrate “continuous compliance with the GDPR” [28]. It is especially vital that each FL participant carefully documents each training data set because of the strict legal obligations to ensure the accuracy of data sets [28]. In addition, “clear protocols should be established specifying which requirements the training data should meet, in light of (among others) the purpose and target population to which the federated learning model will be applied” [28]. “Further interdisciplinary research should be devoted to investigating which measures are suitable and recommended for adoption into large machine learning environments, such as the ones in which federated learning is typically intended to be used” [28].

The second problem of GDPR obligations is the so-called black box effect, which is inherent in all machine learning systems and violates the principle of transparency. The regulator has recognized this. The proposal of the European Commission for the regulation of AI presented on April 21, 2021, raises the question of what specific measures must be taken to ensure the transparency and interpretability of (high-risk) AI systems. However, the proposed regulation unfortunately does not provide an answer to uncertainty regarding transparency [67]. In the meantime, the GDPR supervisory board is considering relaxing the requirement for AI or machine learning mechanisms “by accepting a *general explanation* as an indication of how and what personal data is going to be processed” [22]. This makes the implementation of FL more manageable as it means that, for FL systems, the right to be informed (articles 13-15 of the GDPR) can be fulfilled by providing a general explanation of the FL process in the terms and conditions. The relevant privacy information could then be agreed to by all parties: the processing purpose as “building a global ML model,” the retention period as “retention for a single training round,” and the parties with access to the data as “only the coordinating central party” [22]. “The explanation of the workings of the federated learning model can be achieved by elaborating on how a defined input can lead to a particular output” [35]. With a growing privacy awareness among patients, models need to be carefully designed, and the implications when using them need to be clear [35].

### Strengths and Limitations

We summarized a wide range of subtly different arguments and conclusions in a highly condensed form. In consequence, our review is (by nature) very heterogeneous and relevant to several disciplines.

Nonetheless, the diversity of the relevant publications leads to several methodological challenges. However, owing to the novelty of the research topic, we decided to focus on inclusiveness rather than on specificity. Thus, although we are aware that the lack of quality assessment of the included publications is a limitation of our review, in contrast, it allows us to provide a broad overview of all existing literature. In addition, a fair quality evaluation would have been impossible because of the lack of homogeneity in the included publications. This diversity also led to difficulties in data extraction. Nonetheless, by extracting information through 2 independent researchers, we increased the chance of completeness of our findings. Finally, the aforementioned ultimately renders our results and conclusions theoretical rather than empirical.

Finally, our legal assessment is considerably limited by a lack of court decisions on the subject. Legal facts are ultimately created in judicial decisions, which are absent thus far, to the best of our knowledge.

### Conclusions

We performed a scoping review and identified what we consider to be the most important intersections between data protection legislation and FL techniques. We found that only the combination of FL with SMPC and DP has made the technology sufficiently secure to satisfy the requirements of the GDPR, thereby enabling the use of powerful machine learning tools even for systems that process sensitive personal data such as health data. However, 2 substantial challenges remain: one technical and one legal. In the technical domain, developers must face the need for (and legal prescription of) data protection head on, whereas the negative effects on model utility of techniques such as DP need to be addressed to unlock the full potential of FL systems. In the legal domain, even though the GDPR provides for clauses in terms and conditions that permit the use of FL, the legal hurdles to onboarding of data subjects and “gray areas” nonetheless remain onerous and in urgent need of legislative clarification.

## Appendix

Multimedia Appendix 1 Search strings and quantity for each database searched.

Multimedia Appendix 2 List of individual sources of evidence and assignment to the research questions addressed.

## Abbreviations

AI: artificial intelligence
DP: differential privacy
FL: federated learning
GDPR: General Data Protection Regulation
PRISMA-ScR: Preferred Reporting Items for Systematic Reviews and Meta-Analyses extension for Scoping Reviews
RQ: research question
SMPC: secure multiparty computation
