# Supplementary material for: Federated Machine Learning, Privacy-Enhancing Technologies, and Data Protection Laws in Medical Research: Scoping Review
Source: J Med Internet Res. 2023 Mar 30;25:e41588. doi: 10.2196/41588 (PMC10131784; doi:10.2196/41588)
Supplement: Multimedia Appendix 1 [file jmir_v25i1e41588_app1.docx]

# **Multimedia Appendix 1**

***Table S1*: Search strings and quantity of Beck-online**

| Search string/filter 1 | ((federated learning) AND (“Datenschutzrecht” OR (Data protection)) |
| --- | --- |
| Result quantity | 22 |
| Selected papers | 2 |
| Search string/filter 2 | ((differential privacy) OR ((secure) multiparty computation)) |
| Result quantity | 121 |
| Selected papers | 3 |
| Total selected | **5** |

***Table S2*: Search strings and quantity of SSRN**

| Search string/filter 1 | ((federated learning) AND ((data protection) OR (privacy protection)) |
| --- | --- |
| Result quantity | 5 |
| Selected papers | 1 |
| Search string/filter 2 | ((federated learning) AND ((differential privacy) OR (secure multiparty computing)) |
| Result quantity | 2 |
| Selected papers | 1 |
| Total selected | 1 |

***Table S3*: Search strings and quantity of ScienceDirect**

| Search string/filter 1 | ((federated learning) AND ((data protection) AND (regulation) OR (privacy protection)) AND (Open Access [Filter]) AND (Years 2016-2022 [Filter])) |
| --- | --- |
| Result quantity | 162 |
| Selected papers | 1 |
| Search string/filter 2 | ((federated learning) AND ((differential privacy) OR (secure multiparty computing)) NOT ((data protection) OR (regulation) OR (privacy protection)) AND (Open Access [Filter]) AND (Years 2016-2022 [Filter])) |
| Result quantity | 4 |
| Selected papers | 1 |
| Total selected | 2 |

***Table S4*: Search strings and quantity of ArXiv**

| Search string/filter 1 | ((federated learning) AND (data protection) AND (personal data)) |
| --- | --- |
| Result quantity | 38 |
| Selected papers | 8 |
| Search string/filter 2 | ((federated learning) AND ((differential privacy) OR (secure multiparty computing)) NOT ((data protection) OR (personal data))) |
| Result quantity | 234 |
| Selected papers | 14 |
| Total selected | 22 |

***Table S5*: Search strings and quantity of Google Scholar**

| Search string/filter | ((federated learning) AND ((data protection) OR (privacy protection) OR (GDPR-compliance) OR (DSGVO)) AND (Years 2016-2022 [Filter])) |
| --- | --- |
| Result quantity | 4,140 |
| Selected papers | 12 |
| Search string/filter 2 | ((federated learning) AND ((differential privacy) OR (secure multiparty computing)) NOT ((data protection) OR (privacy protection) OR (GDPR-compliance) OR (DSGVO)) AND (Years 2016-2022 [Filter])) |
| Result quantity | 1,770 |
| Selected papers | 14 |
| Total selected | 26 |
