# Supplementary material for: Federated Machine Learning, Privacy-Enhancing Technologies, and Data Protection Laws in Medical Research: Scoping Review
Source: J Med Internet Res. 2023 Mar 30;25:e41588. doi: 10.2196/41588 (PMC10131784; doi:10.2196/41588)
Supplement: Multimedia Appendix 2 [file jmir_v25i1e41588_app2.docx]

**Multimedia Appendix 2**

***Table S1*: List of individual sources of evidence and assignment to research questions addressed.**

RQ= Research Question; GDPR= General Data Protection Regulation

**RQ1:** Are the local or global models used in federated learning “personal data” as defined in Article 4 (1) GDPR? What could be the consequences in legal terms? **RQ2:** What are the roles of FL model service providers and training participants in relation to the roles set out in Chapter 4 of the GDPR (Controller and Processor)? **RQ 3:** Who controls the (raw) training data used to train the local models in decentralized federated learning? Who controls the model updates aggregated into the global model? **RQ 3.1:** How are the raw training data and model updates secured? **RQ 3.2:** Which basic principles of data protection law and rights of data subject must be observed during training? **RQ 4:** Does the use of Secure Multiparty Computation and/or differential privacy change the previous legal assessment?

| **Authors** | **Year** | **Title** | **Journal/**  **Publisher** | **RQ1** | **RQ2** | **RQ3** | **RQ 3.1** | **RQ 3.2** | **RQ4** |
| --- | --- | --- | --- | --- | --- | --- | --- | --- | --- |
| Aichroth, et al. | 2020 | Anonymisierung und Pseudonymisierung von Daten für Projekte des maschinellen Lernens: Eine Handreichung für Unternehmen | bitkom | x | .. | .. | x | x | x  yes |
| Asad, et al. | 2020 | A Critical Evaluation of Privacy and Security Threats in Federated Learning | mdpi | x | .. | .. | .. | .. | .. |
| Baracaldo, et al. | 2021 | Accountable Federated Machine Learning – wie Modelle ohne Datenzugriff überprüft werden | ibm | x | .. | .. | .. | x | .. |
| Blanco-  Justicia, et al. | 2021 | Achieving security and privacy in federated learning systems: Survey, research challenges and future directions | ScienceDirect | x | .. | .. | .. | .. | .. |
| Boenisch, et al. | 2021 | When the Curious Abandon Honesty: Federated Learning Is Not Private | arXiv | .. | .. | .. | .. | .. | x  no |
| Bonawitz, et al. | 2021 | Federated Learning and Privacy | ACM digital library | .. | .. | .. | .. | .. | x  yes |
| Bonura, et al. | 2022 | Increasing Trust within a Data Space with Federated learning, in Data Spaces: Design, Deployments, and Future Directions | musketeer | x | .. | .. | .. | .. | .. |
| Borutta, et al. | 2020 | Strategiepapier der EU-Kommission zur Künstlichen Intelligenz (KI) | Newsdienst MMR-Aktuell  (CH.Beck) | .. | .. | .. | .. | .. | x  inconclusive |
| Brundage, et al. | 2020 | Toward Trustworthy AI Development: Mechanisms for Supporting Verifiable Claims | arXiv | x | .. | .. | .. | .. | .. |
| Byrd | 2021 | Responsible machine learning: supporting privacy preservation and normative alignment with multi-agent simulation | smartech | .. | .. | .. | .. | .. | x  yes |
| Campos, et al. | 2022 | Evaluating Federated Learning for intrusion detection in Internet of Things: Review and challenges | ScienceDirect | x | .. | .. | .. | .. | .. |
| Chamikara, et al. | 2022 | Local Differential Privacy for Federated Learning in Industrial Settings | arXiv | x | .. | .. | .. | .. | .. |
| Choudhury, et al. | 2020 | Anonymizing Data for Privacy-Preserving Federated Learning | arXiv | x | .. | .. | .. | .. | .. |
| Culea, et al. | 2021 | A Survey of Two Open Problems of Privacy-Preserving Federated Learning: Vertically Partitioned Data and Verifiability | TUDelft | .. | .. | .. | x | .. | .. |
| Deutscher Bundestag | 2021 | Minutes of the 73rd session of the digital agenda committee, no. 19/73 | Deutscher Bundestag | x | .. | .. | .. | .. | x  no |
| Do, et al. | 2020 | Design and Analysis of a GDPR-Compliant Federated Machine Learning System | Computer Science | x | .. | .. | x | x | .. |
| Ebers, et al. | 2021 | Der Entwurf für eine EU-KI-Verordnung: Richtige Richtung mit Optimierungsbedarf | Recht Digital (CH.Beck) | .. | .. | .. | .. | .. | x  no |
| Filip et al. | 2021 | An overview of hybrid approaches in Horizontal Federated Learning | TUDelft | .. | .. | .. | x | .. | .. |
| Geiping, et al. | 2020 | Inverting Gradients – How easy is it to break privacy in federated learning? | NeurIPS Proceedings | x | .. | .. | .. | .. | .. |
| Goldblum | 2021 | Dataset Security for Machine Learning: Data Poisoning, Backdoor Attacks | arXiv | .. | .. | .. | .. | .. | x  no |
| Gutierrez, et al. | 2020 | How You Can Use Federated Learning for Security & Privacy | odsc | x | .. | .. | .. | .. | .. |
| Hatamizadeh, et al. | 2021 | Towards Understanding the Risks of Gradient Inversion in Federated Learning | research-  square | .. | .. | .. | .. | .. | x  no |
| Hartmann | 2018 | Federated Learning | Freie Universität Berlin | x | .. | .. | .. | .. | .. |
| Homescu | 2020 | Personalized treatment through biosensors and machine learning ML | SSRN | x | .. | .. | .. | .. | .. |
| Hu, et al. | 2021 | Federated Leaning: A Distributed Shared Machine Learning Method | hindawi | x | .. | .. | .. | .. | .. |
| Hu, et al. | 2022 | Federated Learning with Sparsified Model Perturbation: Improving Accuracy under Client-Level Differential Privacy | arXiv | .. | .. | .. | .. | .. | x  no |
| Kanagavelu, et al. | 2020 | Two-Phase Multi-Party Computation Enabled Privacy-Preserving Federated Learning | arXiv | x | .. | .. | .. | .. | x  no |
| Kaulartz, et al. | 2020 | Rechtshandbuch Artificial Intelligence und Machine Learning | C.H.Beck | x | .. | .. | .. | .. | x  yes |
| Kaulartz | 2019 | Datenschutz-Compliance bei KI am Beispiel Federated Learning | CMS Deutschland | x | x | .. | .. | .. | x  yes |
| Kurupathi, et al. | 2020 | Survey on Federated Learning Towards Privacy Preserving AI | csitcp | x | .. | .. | .. | x | .. |
| Lee, et al. | 2020 | Federated Leaning on Clinical Benchmark Data: Performance Assessment | jmir | x | .. | x | .. | .. | .. |
| Lepri et al. | 2021 | Ethical machines: The human-centric use of artificial intelligence | iScience | x | .. | .. | .. | .. | x  yes |
| Liu, et al. | 2020 | Learn to Forget: User-Level Memorization Elimination in Federal Learning | researchgate | .. | .. | .. | x | x | .. |
| Lowy, et al. | 2021 | Private Federated Learning Without a Trusted Server: Optimal Algorithms for Convex Losses | arXiv | x | .. | .. | .. | .. | .. |
| Lyu, et al. | 2022 | Privacy and Robustness in Federated Learning: Attacks and Defenses | arXiv | x | .. | .. | .. | .. | .. |
| Lyu, et al. | 2020 | Threats to Federated Learning: A Survey | arXiv | x | .. | .. | .. | .. | .. |
| Ma, et al. | 2021 | When Federated Learning Meets Blockchain: A New Distributed Learning Paradigm | arXiv | x | .. | .. | .. | .. | .. |
| More, et al. | 2022 | SCOTCH: An Efficient Secure Computation Framework for Secure Aggregation | arXiv | x | .. | .. | .. | .. | x  no |
| Naseri, et al. | 2021 | Local and Central Differential Privacy for Robustness and Privacy in Federated Learning | arXiv | .. | .. | .. | .. | .. | x  no |
| Ni, et al | 2021 | Federated Learning Model with Adaptive Differential Privacy in Medical IoT | hindawi | x | .. | .. | .. | .. | .. |
| Orekondy, et al. | 2020 | Gradient-Leaks: Understanding and Controlling Deanonymization in Federated Learning | arXiv | x | .. | .. | .. | .. | .. |
| Paal, et al. | 2021 | DSGVO BDSG | CH.Beck | .. | .. | .. | .. | .. | x  yes |
| Pentyala, et al. | 2022 | Training Differentially Private Models with Secure Multiparty Computation | arXiv | .. | .. | .. | .. | .. | x  no |
| Puschky | 2022 | Federated Learning – eine datenschutzfreundliche Methode zum Trainieren von KI-Modellen? | Newsdienst ZD-Aktuell  (CH.Beck) | .. | .. | .. | .. | .. | x  yes |
| Ribero, et al. | 2020 | Federating Recommendations Using Differentially Private Prototypes | arXiv | x | .. | .. | .. | .. | .. |
| Reddy, et al. | 2020 | Security and Privacy Preserving Deep Learning | arXiv | .. | .. | .. | .. | .. | x  no |
| Rossello, et al. | 2021 | Data protection by design in AI? The case of federated learning | Computerrecht | x | x | x | x | x | x  yes |
| Smith M.D. | 2021 | A Study on Federated Learning Systems in Healthcare | digital maag | .. | .. | .. | .. | .. | x  yes |
| Truex, et al. | 2019 | A Hybrid Approach to Privacy-Preserving Federated Learning | arXiv | .. | .. | .. | .. | .. | x  no |
| Truong, et al. | 2021 | Privacy preservation in federated learning: An insightful survey from the GDPR perspective | Computers & Security | x | x | x | x | x | x  yes |
| Ulhag, et al. | 2020 | Covid-19 Imaging Data Privacy by Federated Learning Design: A Theoretical Framework | arXiv | x | .. | .. | .. | .. | .. |
| Yang, et al. | 2021 | An Accuracy-Lossless Perturbation Method for Defending Privacy Attacks in Federated Learning | arXiv | x | .. | .. | .. | .. | .. |
| Yang, et al. | 2019 | Federated Machine Learning: Concept and Applications | ACM digital library | .. | .. | .. | .. | .. | x  no |
| Zhao, et al. | 2020 | Local Differential Privacy based Federated Learning for the Internet of Things | arXiv | x | .. | .. | .. | .. | .. |
| Zhao, et al. | 2021 | Utility Optimization of Federated Learning with Differential Privacy | hindawi | x | .. | .. | .. | .. | .. |
| Zheng, et al. | 2021 | FL-Market: Trading Private Models in Federated Learning | arXiv | x | .. | .. | .. | .. | .. |
